# Treated wastewater effluent increases pharmaceutical concentrations and alters benthic microbial communities in streams

**DOI:** 10.3389/fmicb.2025.1649739

**Published:** 2025-08-26

**Authors:** Benjamin Lorentz, Madeleine Rauhauser, Ryan T. Krantz, Daniel D. Snow, John J. Kelly

**Affiliations:** ^1^Department of Biology, Loyola University Chicago, Chicago, IL, United States; ^2^School of Natural Resources, University of Nebraska, Lincoln, NE, United States

**Keywords:** wastewater, effluent, stream, pharmaceuticals, antibiotics, microbial community, bacteria, resistance

## Abstract

Wastewater treatment plant (WWTP) effluent can be a point source of pharmaceuticals and personal care products (PPCPs) to surface waters, and these biologically active compounds have the potential to select for resistant traits and taxa within aquatic microbial communities. The goals of this study were to determine if WWTP effluent is a point source of PPCPs to urban streams; to determine if effluent inputs affect benthic microbial community composition; and to determine if effluent inputs increase the abundance of antibiotic resistance determinants within benthic microbial communities. We collected water and sediment from three streams in the Chicago metro area: two urban streams that receive WWTP effluent and one rural stream that does not receive effluent. We quantified concentrations of a suite of 45 common PPCPs in water samples from each stream, including sites upstream and downstream of effluent inputs to the urban streams, analyzed benthic bacterial community composition, and quantified the abundance of *intI1*, a gene linked to antibiotic resistance. A stream receiving 80% of its flow from effluent showed higher concentrations of ten PPCPs, including several antibiotics, downstream of the effluent input, as well as decreased abundance of photosynthetic organisms and shifts in bacterial community composition, implicating effluent as the driver of these changes. We did not observe differences between upstream and downstream sites in a stream receiving only 13% of its flow from effluent. The *intI1* gene did not differ in abundance within streams in response to effluent input, but *intI1* abundance and PPCP concentrations were higher in the urban streams than in the rural stream. These results indicate that watershed-scale anthropogenic impacts were the driver of *intI1* abundance and that non-point sources contributed to PPCP pollution.

## 1 Introduction

Domestic wastewater refers to water released from residences and businesses that has been contaminated through human activities such as restroom usage, washing, bathing, food preparation, and laundry. The release of large volumes of untreated domestic wastewater to the environment can have negative impacts on aquatic ecosystems due to the nutrient content of wastewater and the potential presence of pathogens (Rittmann and McCarty, 2000). Wastewater treatment plants (WWTPs) are the most common method used to treat domestic wastewater in urban and suburban areas of the United States, serving over 75% of the population (US EPA, 2009). WWTPs are designed to reduce the nutrient and pathogen content of wastewater prior to its release to the environment (Rittmann and McCarty, 2000). WWTPs frequently release treated water (referred to as effluent) to surface waters, including streams and rivers (US EPA, 2009), and WWTP effluent can be a major source of flow to these ecosystems (Brooks et al., 2006). For example, WWTP effluent accounts for >50% of the flow of more than 900 rivers in the United States (Rice and Westerhoff, 2017). The contribution of effluent to stream flow can be especially significant in urban areas with high population densities; for example, effluent accounts for >70% of the annual flow of the Chicago River (Illinois Department of Natural Resources, 2011). Despite treatment, WWTP effluent can have negative effects on streams and rivers, including increased nutrient loading (Waiser et al., 2011a) and eutrophication (Gücker et al., 2006).

Domestic wastewater can also contain pharmaceuticals and personal care products (PPCPs) that are released into wastewater through normal use, including stimulants, analgesics, antibiotics, antiseptics, antihistamines, and other medications (Daughton and Ternes, 1999; Hedgespeth et al., 2012). For example, between 30% and 90% of ingested antibiotics are excreted unchanged by humans (Sarmah et al., 2006), and compounds used externally (e.g., cosmetics, antiseptics, topical steroids, topical antihistamines) enter wastewater via washing and bathing. WWTPs are not generally designed to remove PPCPs, although some are removed incidentally at widely varying rates (Aga, 2007; Watkinson et al., 2007), so WWTP effluent can be a point source of many PPCPs to the environment (Hoellein et al., 2017; Waiser et al., 2011b). For commonly used PPCPs that are not completely removed during wastewater treatment, WWTPs continuously deliver these compounds to the receiving ecosystem, resulting in their pseudo-persistence in surface waters throughout the United States (Heberer, 2002; Kolpin et al., 2002).

PPCPs are biologically active compounds that can disrupt ecological processes in aquatic ecosystems (Richmond et al., 2017) and interact with aquatic biota, including microorganisms (Akiyama and Savin, 2010; Rosi-Marshall and Kelly, 2015). The experimental addition of amphetamines and the antidepressants fluoxetine and citalopram to artificial stream mesocosms resulted in decreases in gross primary production and community respiration in microbial biofilms (Lee et al., 2016; Richmond et al., 2016), while a mixture of PPCPs resulted in decreased algal biomass (Corcoll et al., 2015). Other studies have demonstrated decreases in respiration rates and shifts in bacterial community composition in biofilms experimentally exposed to a range of PPCPs, including antibiotics, antihistamines, and stimulants (Costello et al., 2016; Rosi et al., 2018; Rosi-Marshall et al., 2013). While these studies have identified possible effects of PPCPs on stream microbial communities, they were based on manipulative experiments using either artificial streams (Richmond et al., 2016, 2019) or artificial substrates (Rosi et al., 2018; Rosi-Marshall et al., 2013), and generally included high PPCP concentrations. Field-based studies of native microbial communities under real-world PPCP exposure scenarios are more limited (Hagberg et al., 2021). This study was designed to address this knowledge gap by assessing the potential of WWTP effluent to serve as a point source of PPCPs and impact bacterial community composition in the field.

PPCPs in the environment can also create selection pressure on microbial communities and select for genes that confer antimicrobial resistance (ARGs). This selection pressure can increase the abundance of ARGs within the environmental resistome and potentially contribute to the spread of antibiotic resistance to pathogens (Allen et al., 2010; Rosi-Marshall and Kelly, 2015). In this study we quantified the abundance of the clinical class 1 integrase gene, *intI1*, which is commonly linked to genes conferring resistance to antibiotics, disinfectants, and heavy metals (Liebert et al., 1999; Partridge et al., 2001). Because of its frequent association with ARGs and its propensity to propagate across diverse bacterial taxa, *intI1* abundance has been used as an indicator of bacterial community resistance to a variety of anthropogenic stressors (Gillings et al., 2015) and previous studies have documented increased abundance of *intI1* in environments impacted by anthropogenic pollution, including wastewater effluent (for review see Gillings et al., 2015).

The goals of this study were to (1) determine if WWTP effluent is a point source of PPCPs to streams in the Chicago metro area, (2) determine if WWTP effluent affects benthic microbial community composition, and (3) determine if WWTP effluent increases the frequency of antibiotic resistance determinants in benthic microbial communities. To achieve these goals, we collected water and sediment from three streams in the Chicago metro area: two urban streams which receive WWTP effluent and one rural stream that does not receive effluent. In the two urban streams we collected water and sediment from sites upstream and downstream of the effluent input points. We quantified concentrations of a suite of 45 common PPCPs in water samples from each site, analyzed benthic bacterial community composition using high-throughput amplicon sequencing (Rout et al., 2021), and quantified the abundance of *intI1* using quantitative polymerase chain reaction (qPCR). The two effluent-receiving streams differed in the relative contribution of effluent to stream flow, so we hypothesized that the high effluent stream would have higher concentrations of PPCPs at its downstream site resulting in greater effects on microbial communities and *intI1* abundance and that these effects would be less significant or absent for the low effluent stream. Furthermore, we hypothesized that PPCP concentrations would be lower in the rural stream compared to the urban streams, resulting in comparatively lesser impacts on microbes and the resistome. By comparing responses to elevated PPCP concentrations in the two urban streams with the rural stream, we assessed watershed-scale impacts of anthropogenic pharmaceutical contamination on aquatic microbiota.

## 2 Methods

### 2.1 Study sites

The control site (CONTROL) was located on Nippersink Creek, a rural stream located in McHenry County, IL which has minimal urbanization in its watershed. Nippersink Creek has a drainage area of 5,095 ha that is 63.1% agricultural, 20.7% open land, 7.8% residential, 2.1% vacant, and 0.1% industrial (www.nippersink.org). There are no WWTPs or combined sewer overflows (CSOs) on Nippersink Creek upstream of the sampling site. Water and sediment were collected from one site on Nippersink Creek (N 42° 5′ 6.6′‘ W 88° 0′ 40.76″; Supplementary Figure S1) on 11/4/2019.

Salt Creek, an urban stream in DuPage County, IL, was chosen to represent a stream with a low proportion of its flow contributed by WWTP effluent (LO-EFF). Salt Creek has a drainage area of 3,948 ha that is 49.9% residential, 16.8% open land, 11.2% commercial, 6.3% industrial, 4.1% transportation, and 0.3% agricultural (www.drscw.org/watershed-descriptions/). Salt Creek receives treated wastewater effluent from the Elmhurst WWTP, which has an average flow of 7.03 million gallons per day, accounting for ~13% of the flow of Salt Creek downstream of the WWTP (McCormick et al., 2016). Water and sediment were collected from two sites on Salt Creek on 11/6/2019, one 200 meters upstream of the WWTP effluent input point (LO-EFF-UP; N 41° 2′ 58.16″ W 87° 7′ 33.23″; Supplementary Figure S1) and the other 256 meters downstream of the effluent input point (LO-EFF-DN; N 41° 2′ 43.76″ W 87° 7′ 297″; Supplementary Figure S1).

Spring Brook, an urban stream in DuPage County, IL, was chosen to represent a stream with a high proportion of its flow contributed by WWTP effluent (HI-EFF). Spring Brook has a drainage area of 1,991 ha that is 46.8% residential, 30.6% open land, 8.8% transportation, 2.0% commercial, 0.5% agricultural, and 0.0% industrial (https://epa.illinois.gov/). Spring Brook receives treated wastewater effluent from the Wheaton Sanitary District WWTP, which has an average flow of 7.39 million gallons per day, accounting for ~80% of the flow of Spring Brook downstream of the WWTP (McCormick et al., 2016). Water and sediment were collected from two sites on Spring Brook on 11/6/2019, one 750 meters upstream of the WWTP effluent input point (HI-EFF-UP; N 41° 0′ 52.66″ W 88°8′ 24.06″; Supplementary Figure S1) and the other 100 meters downstream of the effluent input point (HI-EFF-DN; N 41° 0′ 33.92″ W 88°8′ 48.63″; Supplementary Figure S1).

### 2.2 Sample collection

The following sampling was conducted at each of the 5 field sites. Five replicate 20 mL water samples were collected using a 50 mL syringe and filtered on-site with 0.2 μm syringe filters (Thermo Fisher Scientific, Waltham, MA) into sterile scintillation vials which were transported on ice in a cooler to the lab where they were stored at −20°C for subsequent nutrient chemistry analysis. Three replicate 1L water samples were collected in amber glass bottles with Teflon-lined lids and transported on ice to the lab where they were stored at 4°C for subsequent PPCP analysis. Finally, five replicate sediment samples were collected from the stream using a shovel. Sediment was passed through a 4 mm sieve into a plastic tub, homogenized by mixing, and subsampled into a sterile 90 mL plastic specimen cup (Parter Medical, Carson CA). Replicate sediment samples were collected from the stream benthos at locations at least 1 m apart. The shovel, sieve, and tub were rinsed with stream water between replicates and were sterilized with ethanol between sites. The specimen cups were stored on ice for transport to the lab where they were stored at 4°C overnight. The next day five 0.5 mL subsamples of each sediment sample were transferred to 2 mL microcentrifuge tubes and stored at −20°C for subsequent DNA extraction. Approximately 10 g of the remaining sediment was used for quantification of organic matter content. At each field site, water temperature, dissolved oxygen, specific conductance, total dissolved solids, salinity, pH, turbidity, and chlorophyll-a and phycocyanin concentrations were measured using a YSI ProDSS multiparameter water quality meter (YSI Yellow Springs, OH). Replicate readings (*n* = 3) of these parameters were taken at each of the field sites.

### 2.3 Nutrient chemistry and sediment organic matter

We quantified dissolved soluble reactive phosphorus (SRP), ammonium (${\text{NH}}_{4}^{+}$), and nitrate (${\text{NO}}_{3}^{-}$) in water samples with a Seal Analytical Autoanalyzer (Seal Analytical, Mequon WI) using the antimonyl tartarate, phenol hypochlorite, and Cd reduction methods, respectively (APHA, 1998). These analyses were completed within 10 weeks of collection. We followed quality control and assurance checks recommended by the manufacturer (Seal Analytical) including equipment blanks, carryover tests, and drift correction. All standard curves showed r^2^ ≥ 0.999. Organic matter content of the sediment samples was calculated by loss on ignition at 500°C (Ball, 1964).

### 2.4 Pharmaceutical analysis

Water samples for PPCP analysis were loaded onto solid phase extraction (SPE) cartridges (Oasis HLB cartridges, Waters Corporation, Milford, MA) within 2 weeks of sample collection. Cartridges were first conditioned with 5–10 mL of methanol and then 5–10 mL of deionized water. Sample water was passed through a 25 mm diameter, 1 um pore size glass fiber filter and then through the SPE cartridge at a rate of 1 drop per second until approximately 300 mL was passed through the filter, with the specific volume passed recorded for each sample. Cartridges were stored at −20°C and then sent to the Water Sciences Laboratory at the University of Nebraska for analysis. There, 100 μL of 0.1 ng/μL surrogate compounds (13C3-Atrazine, 13C3-DEA, Orphenadrine HCl, Oleandomycin) were spiked onto the cartridges and allowed to dry. Blank samples were prepared by extracting distilled deionized water onto SPE cartridges. A fortified blank was prepared by spiking 100 μL of 0.1 ng/μL analyte spike into one of the blanks. Samples were then eluted using 10 mL of acetonitrile. Once the elution was complete, samples were evaporated under a stream of dry nitrogen until they measured approximately 500 μL. Internal standards were added in a 100 μL aliquot of 0.1 ng/μL solution (3C,15N-Acetaminophen, 13C3,15N-Ciprofloxacin, 13C3-Caffeine, 13C3-Trimethoprim, 13C6-Carbamazepine, 13C6-Sulfamethazine-phenyl, 13C6-Sulfamethoxazole, 13C6-Thiabendazole, 13C-N-Methylerythromycin-d3, Cotinine-d3, and Fluoxetine-d6). After this addition the evaporation continued until approximately 40 μL remained. The samples were then reconstituted using 160 μL of distilled deionized water, vortexed, and transferred to vials with silanized glass inserts. Extracts were analyzed using an Aquity ultra high-pressure chromatography system and multiple reaction monitoring on a Xevo TQS micro tandem mass spectrometer using a UniSpray^TM^ ionization source (Waters Corporation, Milford, MA). Source and instrument conditions are provided in Supplementary Table S1. Quantitation and confirming ion transitions were selected based on USEPA Method 1694 (Ferrer et al., 2010). Gradient separation was achieved with mobile phase solvents consisting of C: 0.3% (v/v) formic acid and D: 0.1% (w/v) ammonium formate in 1:1 methanol:acetonitrile at a flow rate of 0.6 mL/min. Initial mobile phase conditions are 100:0 C/D, gradually increase to 70:30 C/D at 1 min and 30:70 C/D at 5 min. Hold for 0.5 min and switch to 0:100 C/D. Hold for 3.5 min before switching back to the original condition 100:0 C/D at 9 min. Total run time is 12 min per sample. An Acquity Premier BEH C18 VanGuard Fit reverse phase UPLC column (50 mm × 2.1 mm × 1.7 μm) was employed for the chromatographic separation of analytes at a controlled temperature of 40°C (Waters Corporation, Milford, MA). MRM transitions, retention times, cone voltages, and collision energies are included in Supplementary Table S2. Instrument detection limits, estimated as 3 times the noise level, and method detection limits following US EPA protocols are listed in Supplementary Table S3. Quality controls included analysis of laboratory method blanks and fortified blanks.

### 2.5 Statistical analysis of stream physical and chemical data

Physical, chemical, and PPCP data from each site were statistically analyzed in R (v3.6.1). We assessed normality of these data with the Shapiro-Wilk test (Shapiro and Wilk, 1965). Normally distributed data were analyzed via one-way analysis of variance (ANOVA; Fisher, 1992) according to site. Statistically significant ANOVA results (*p* < 0.05) were further analyzed using Tukey's Honestly-Significant-Difference Test (Tukey, 1949) to determine significant pairwise differences. Non-normally distributed data were analyzed via the non-parametric Kruskal-Wallis rank sum test (Kruskal and Wallis, 1952) according to site. Statistically significant Kruskal-Wallis results were further analyzed using Dunn's test (Dunn, 1961) to determine significant pairwise differences.

### 2.6 DNA extraction

DNA was extracted from all sediment samples using the Qiagen DNeasy Power Soil Extraction Kit (Qiagen, Inc., Hilden, Germany) and successful extraction was confirmed with agarose gel electrophoresis. Two extraction kits without samples were run as contamination controls and produced no visible bands on agarose gels. Extracted DNA was quantified with a NanoDrop 8000 spectrophotometer (Thermo Fisher Scientific, Waltham, MA).

### 2.7 Quantification of clinical integrase gene *intI1*

To create a standard for qPCR analysis of the class 1 integrase gene *intI1*, this gene was amplified from DNA extracts from each replicate sediment sample from each field site (total of 25 samples) via standard PCR using primers *intI1* LC1 and *intI1* LC5, which were designed to target clinical *intI1* genes (Barraud et al., 2010). Thermocycling parameters were as follows: denaturing at 95°C for 5 min, 35 cycles consisting of 30 s denaturing at 95°C, 45 s annealing at 54°C, and 30 s extension at 72°C, and a final 7 m extension at 72°C. Amplification was confirmed via agarose gel electrophoresis. Amplicons from all samples were pooled and cloned using the Invitrogen TOPO TA Cloning kit (Thermo Fisher Scientific, Waltham, MA) and NEB 10-beta high efficiency competent *Escherichia coli* cells (New England BioLabs, Ipswich, MA). Plasmids were isolated from 5 randomly selected colonies using the Invitrogen Pure Link Quick Plasmid Miniprep kit (Thermo Fisher Scientific, Waltham, MA). Successful insertion of the *intI1* amplicon was confirmed by PCR using primers M13F and M13R and agarose gel electrophoresis. Plasmids were sequenced by Plasmidsaurus (South San Francisco, CA) and identity of the inserts was confirmed as *intI1 by* comparison to the NCBI database using BLAST. Insert sequences were aligned using T-COFFEE (Notredame et al., 2000), which revealed 100% identity among amplicons with the exception of one single nucleotide polymorphism. All five plasmid samples were pooled and DNA concentration was determined using a NanoDrop 8000 spectrophotometer. A standard dilution series ranging from 10^8^ to 10^2^ *intI1* gene copies μl^−1^ was prepared.

To create a standard for qPCR analysis of the 16S rRNA gene, *Staphylococcus aureus* (ATCC 700699) was cultured overnight in Brain Heart Infusion Broth and DNA was extracted using the DNeasy UltraClean Microbial Kit (Qiagen). *S. aureus* DNA concentration was determined using a NanoDrop 8000 spectrophotometer and a standard dilution series ranging from 10^8^ to 10^2^ 16S rRNA gene copies μl^−1^ was prepared.

QuantStudio 3 Real-Time PCR System (Thermo Fisher Scientific, Waltham, MA) was used to quantify *intI1* and 16S rRNA gene copy numbers in DNA extracts from each replicate sample from each site (total of 25 samples). Primers *intI1*-LC1 and *intI1*-LC5 were used to amplify the *intI1* gene (Barraud et al., 2010) and primers 515F and 806R were used to amplify the 16S rRNA gene (Caporaso et al., 2012). Reaction mixtures included 12.5μL QuantiTect SYBR green PCR master mix (Qiagen, Hilden, Germany). Thermocycling parameters for *intI1* amplification were as follows: denaturing at 95°C for 15 min, 40 cycles consisting of denaturing at 95°C for 45s, annealing at 54°C for 45s, extension at 72°C for 45s, and imaging at 78°C for 12s, followed by melt curve analysis. Cloned *intI1* genes (described above) were used as the quantification standard and each environmental sample was run in triplicate. The *intI1* standard dilution series produced a standard curve with R^2^ = 0.999 and an amplification efficiency of 105%. Thermocycling parameters for 16S rRNA amplification were the same as above with the exception of a 55°C annealing temperature. Genomic DNA from *S. aureus* (described above) was used as the quantification standard for 16S rRNA genes and each environmental sample was run in triplicate. The 16S rRNA standard dilution series produced a standard curve with R^2^ = 0.995 and an amplification efficiency of 84%. Quant Studio Design and Analysis software (Thermo Fisher Scientific, Waltham, MS) was used to determine copy numbers for each gene target in each sample.

The copy number of class 1 integrase gene *intI1* in each sample was normalized to copies of the bacterial 16S rRNA gene in that sample (Gillings et al., 2015). The normalized data were graphed using R (v4.3.1; R Core Team, 2020) and the packages ggplot2 (Wickham, 2016), ggpubr (Kassambara, 2023), and dplyr (Wickham et al., 2023). Normalized *intI1* copy numbers were transformed *a priori* via arcsine square root transformation to better meet the homoscedasticity and normality assumptions of ANOVA. The data were statistically evaluated using Levene's test for homogeneity of variance (Levene, 1960) with the car package (Fox and Weisberg, 2019), then normality was assessed using the Shapiro-Wilk test. Statistical results were compared visually to cell mean vs. cell standard deviation and normal probability plots, respectively. After performing a one-way ANOVA, a *post-hoc* Tukey HSD multiple comparisons test determined significant pairwise differences between group means. A subset containing data from LO-EFF and HI-EFF sites was also evaluated via two-way ANOVA to determine the effects of position relative to the WWTP on *intI1* abundance.

### 2.8 Analysis of bacterial community composition via amplicon sequencing

Bacterial 16S rRNA genes were amplified from DNA extracts from each replicate sample from each site (total of 25 samples) using PCR and primers 515F and 806R, which target the V4 hypervariable region (Caporaso et al., 2012). Successful amplification was confirmed with agarose gel electrophoresis. No bands were observed for kit controls, confirming the kits were not a source of contamination. Amplicons were sequenced in a 2 x 250 paired-end format with the MiSeq platform (Illumina, San Diego, California) by the DNA Services Facility, University of Illinois at Chicago. All sequence data analyzed in this paper can be downloaded from the National Center for Biotechnology Information Sequence Read Archive (NCBI SRA) with accession number PRJNA662915.

Amplicon sequences were processed with mothur V.1.42.2 (Schloss et al., 2009) following the MiSeq Standard Operating Procedure (Kozich et al., 2013). Briefly, paired reads were assembled and demultiplexed, and any sequences with ambiguities or homopolymers >8 bases were removed. Sequences were aligned with the SILVA-compatible alignment database available within mothur. Chimeric sequences were identified with UCHIME (Edgar et al., 2011) and removed. Sequences were classified with the mothur-formatted version of the RDP training set (v.9) and any unknown (i.e., not identified as bacterial), chloroplast, mitochondrial, archaeal, and eukaryotic sequences were removed. We randomly subsampled the entire dataset to 58,826 sequences per sample to avoid biases associated with uneven numbers of sequences across samples (Schloss, 2024). Sequences were then clustered into operational taxonomic units (OTUs, hereafter referred to as species) based on 97% sequence identity. We estimated coverage (Good, 1953) for each sample, and this metric indicated that the average coverage across all samples was 89.7% with a range of 87.4–91.3%. OTUs were identified to the lowest possible taxonomic rank by comparison to the mothur-formatted version of the RDP training set. For OTUs of interest that were not identified to the genus level by mothur (i.e. those labeled unclassified) we used mothur to identify a representative sequence for that OTU and compared the representative sequence to the NCBI 16S rRNA sequence database using Megablast to identify the best match. The effect of site on the relative abundance of the 25 most abundant bacterial orders was assessed with the Kruskal-Wallis and Dunn's tests. Bacterial communities were compared across sites at the species level by calculating dissimilarities for each pair of samples based on the Bray-Curtis index (Bray and Curtis, 1957) and visualizing the resulting dissimilarity matrix using principal coordinates analysis (PCOA) in R (v3.6.1). Statistical significance of differences in communities between sites based on the Bray-Curtis index was assessed by analysis of molecular variance (AMOVA), a nonparametric analog of traditional analysis of variance (Excoffier et al., 1992), which was run in mothur and by PERMANOVA, which was run using the adonis tool in the vegan package of R. Metastats analysis (White et al., 2009) run in mothur was used to identify bacterial species (OTUs) that were differentially abundant between upstream and downstream sites from both the high effluent and low effluent streams and to compare the downstream sites from the two streams.

## 3 Results

### 3.1 Site physical and chemical characteristics

There was a significant effect of site on stream physical and chemical characteristics (Tables 1, 2). These results reveal a general pattern of significant differences in physical and chemical characteristics between the upstream and downstream sites on the high effluent stream (HI-EFF), but no differences between the upstream and downstream sites on the low effluent stream (LO-EFF). For example, sediment organic matter was significantly lower at the HI-EFF downstream site (HI-EFF-DN) compared to the HI-EFF upstream site (HI-EFF-UP), but there was no significant difference in sediment organic matter between LO-EFF downstream (LO-EFF-DN) and upstream (LO-EFF-UP; Table 1). HI-EFF-DN also had significantly higher nitrate and soluble reactive phosphorus (SRP) concentrations and a significantly lower ammonium concentration than HI-EFF-UP, but there were no significant differences in these nutrients between LO-EFF-DN and LO-EFF-UP (Table 1). Finally, there were significant effects of site on water temperature, dissolved oxygen concentration, and turbidity (Table 2). Temperature and dissolved oxygen were both significantly higher and turbidity was significantly lower at HI-EFF-DN compared to HI-EFF-UP, but there were no significant differences in temperature, dissolved oxygen, or turbidity between LO-EFF-DN and LO-EFF-UP. Specific conductance, total dissolved solids, salinity, and pH also differed significantly based on site (Table 2), but there were no significant upstream vs. downstream differences for these parameters within either the HI-EFF or LO-EFF streams. Finally, the physical and chemical characteristics of the CONTROL site were generally within the range of the values observed at the other sites with the exception of turbidity which was significantly higher at CONROL than all of the other sites (Table 2).

**Table 1:** Sediment organic matter and water chemistry at field sites.

**Site**	**Organic matter %^a^**	**${\text{NO}}_{3}^{-}$(mg N L ^−1^)^a^**	**SRP (mg P L ^−1^) ^a^**	**${\text{NH}}_{4}^{+}$ (mg N L ^−1^) ^a^**
CONTROL	0.901 +/– 0.064 a	2.473 +/– 0.036 ab	0.000 +/– 0.000 a	0.147 +/– 0.005 ab
LO-EFF-UP	3.504 +/– 0.127 b	4.422 +/– 0.084 bd	0.393 +/– 0.018 ac	0.155 +/– 0.002 a
LO-EFF-DN	2.343 +/– 0.038 bc	4.969 +/– 0.086 cd	0.449 +/– 0.110 bc	0.168 +/– 0.006 a
HI-EFF-UP	2.363 +/– 0.102 b	2.120 +/– 0.012 a	0.000 +/– 0.000 a	0.159 +/– 0.005 a
HI-EFF-DN	1.085 +/– 0.117 ac	12.627 +/– 1.180 c	0.801 +/– 0.070 b	0.090 +/– 0.002 b
Kruskal-Wallis	*p* < 0.001	*p* < 0.001	*p* < 0.001	*p* = 0.005

^a^Mean values (n = 5) +/- standard error. Different letters within a column indicate significant differences between sites based on Dunn's test (*p* < 0.05).

**Table 2:** Water characteristics measured on site.

**Site**	**Temperature (°C)^a^**	**Dissolved (%)${\text{O}}_{2}^{\text{a}}$**	**Specific conductance (μS/cm^−1^)^a^**	**Total dissolved solids (mg/L^−1^)^a^**	**Salinity (ppt)^a^**	**pH ^a^**	**Turbidity (FNU)^b^**	**Chlorophyll (RFU)^a^**	**Phycocyanin (RFU)^b^**
CONTROL	6.9 +/– 0.033 a	93.5 +/– 0.033 a	721.0 +/– 0.000 a	469.0 +/– 0.333 a	0.35 +/– 0.000 a	7.89 +/– 0.019 a	12.4 +/– 0.067 a	6.10 +/– 0.058 a	0.90 +/– 0.050 a
LO-EFF-UP	8.7 +/– 0.000 abc	91.4 +/– 0.000 bc	942.0 +/– 0.000 ac	612.0 +/– 0.000 ac	0.47 +/– 0.000 ac	7.72 +/– 0.006 abc	8.55 +/– 0.782 b	2.55 +/– 0.050 b	0.57 +/– 0.033 b
LO-EFF-DN	9.1 +/– 0.033 bc	92.0 +/– 0.200 abc	948.7 +/– 0.882 abc	617.0 +/– 0.000 abc	0.47 +/– 0.000 ac	7.75 +/– 0.028 ac	6.53 +/– 1.122 b	2.42 +/– 0.120 b	0.48 +/– 0.044 b
HI-EFF-UP	7.7 +/– 0.000 ab	72.7 +/– 1.139 b	1,102.3 +/−0.333 b	716.3 +/– 0.667 b	0.55 +/– 0.000 b	7.48 +/– 0.000 bc	7.65 +/– 1.000 b	1.25 +/– 0.074 c	0.31 +/– 0.010 c
HI-EFF-DN	15.3 +/– 0.033 c	93.4 +/– 0.088 ac	1,056.3 +/– 0.333 bc	686.3 +/– 0.333 bc	0.53 +/– 0.000 bc	7.44 +/– 0.012 b	1.05 +/– 0.026 c	0.57 +/– 0.017 d	0.09 +/– 0.021 d
Kruskal-Wallis ^c^/ANOVA^d^	*p* = 0.008^c^	*p* = 0.01^c^	*p* = 0.008^c^	*p* = 0.008^c^	*p* = 0.005^c^	*p* = 0.01^c^	*p* = 0.01^d^	*p* ≤ 0.001^d^	*p* ≤ 0.001^d^

^a, b^Mean values (n = 3) +/- standard error. Different letters within a column indicate significant differences between sites based on ^a^Dunn's test (*p* < 0.05) or ^b^Tukey's post-hoc test (*p* < 0.05).

### 3.2 Photosynthetic pigment concentrations

The concentrations of photosynthetic pigments phycocyanin and chlorophyll-a followed the same pattern observed for the physical and chemical characteristics, i.e., significant differences between the upstream and downstream sites on HI-EFF, but no differences observed between the upstream and downstream sites on LO-EFF. Specifically, phycocyanin concentration was significantly lower at HI-EFF-DN compared to HI-EFF-UP, but there was no significant difference between LO-EFF-DN and LO-EFF-UP (Table 2). It was also noteworthy that CONTROL had the highest concentration of phycocyanin, almost 2-fold higher than any other site. The pattern for chlorophyll-a concentration was virtually identical to phycocyanin (Table 2). Chlorophyll-a was significantly lower at HI-EFF-DN compared to HI-EFF-UP, while there was no significant difference between LO-EFF-DN and LO-EFF-UP, and CONTROL had a chlorophyll-a concentration more than two-times higher than any other site.

### 3.3 Pharmaceuticals

PPCP concentrations also followed a similar pattern to the parameters discussed above, i.e., significant differences between the upstream and downstream sites on HI-EFF, but no differences observed between the upstream and downstream sites on LO-EFF. Of the 45 PPCPs included in the analysis, 26 were detected in at least one of the samples (Table 3). The compounds detected at the highest concentrations included several antibiotics (sulfamethoxazole, clarithromycin, azithromycin, erythromycin), caffeine and its metabolite 1,7-dimethylxanthine, pain relievers (acetaminophen, codeine), the antihistamine diphenhydramine, and drugs used to treat high blood pressure (diltiazem) and bipolar disorder (carbamazepine). Sixteen of the detected PPCPs varied significantly in concentration across the field sites, and of those 10 had a significantly higher concentration at HI-EFF-DN compared to HI-EFF-UP, including sulfamethoxazole, diphenhydramine, clarithromycin, and azithromycin (Table 3), indicating that effluent input significantly increased the concentrations of these PPCPs in the HI-EFF stream. Conversely, caffeine and acetaminophen concentrations were significantly lower at HI-EFF-DN compared to HI-EFF-UP, suggesting that non-point sources contributed these PPCPs. There were no significant differences in the concentrations of any of the PPCPs between LO-EFF-DN and LO-EFF-UP (Table 3), indicating that effluent input did not significantly increase the concentrations of any of these PPCPs in the LO-EFF stream. The CONTROL stream had the lowest concentrations of all of the detected PPCPs, including 10 of the 26 PPCPs detected in the study which were not detected at the CONTROL site. The upstream sites of the HI-EFF and LO-EFF streams had higher concentrations of several PPCPs compared to CONTROL, including caffeine and acetaminophen, again suggesting that non-point sources contributed PPCPs to these streams.

**Table 3:** Concentration (ng L^−1^) of PPCP compounds in water collected from field sites.

**Compound^a^**	**CONTROL^b^**	**LO-EFF-UP^b^**	**LO-EFF-DN^b^**	**HI-EFF-UP^b^**	**HI-EFF-DN^b^**	***p* value^c^**
Sulfamethoxazole	19.03 +/– 3.32 a	94.13 +/– 10.47 ab	175.93 +/– 32.48 b	1.97 +/– 0.45 a	614.25 +/– 212.08 b	0.010
Caffeine	36.69 +/– 23.88 a	232.00 +/– 84.11 bc	110.41 +/– 19.45 abc	361.23 +/– 19.52 c	87.65 +/– 23.29 ab	0.055
1,7-Dimethylxanthine	3.80 +/– 3.80 a	216.92 +/– 140.31 c	123.22 +/– 9.24 c	98.71 +/– 65.07 bc	10.14 +/– 10.14 ab	0.037
Diltiazem	1.99 +/– 0.31 ab	85.28 +/– 28.10 bc	87.72 +/– 9.60 bc	0.64 +/– 0.13 a	265.05 +/– 53.39 c	0.012
Acetaminophen	1.70 +/– 0.40 a	94.97 +/– 3.95 bc	123.77 +/– 8.54 c	64.66 +/– 0.73 b	0.00 +/– 0.00 a	0.009
Sulfanilamide	0.00 +/– 0.00 a	23.18 +/– 4.87 ab	26.21 +/– 2.55 b	0.00 +/– 0.00 a	140.26 +/– 20.10 b	0.011
Carbamazepine	1.96 +/– 1.05 a	40.68 +/– 4.43 ab	46.99 +/– 1.41 b	0.43 +/– 0.09 a	78.93 +/– 11.07 b	0.014
Diphenhydramine	0.22 +/– 0.06 a	14.46 +/– 7.78 ab	16.48 +/– 0.85 b	0.20 +/– 0.03 a	123.02 +/– 21.91 b	0.016
Clarithromycin	0.57 +/– 0.14 ab	32.32 +/– 9.52 bc	42.84 +/– 4.76 c	0.18 +/– 0.02 a	54.65 +/– 14.14 c	0.021
Azithromycin	0.02 +/– 0.02 ab	11.97 +/– 3.40 bc	14.84 +/– 1.56 c	0.00 +/– 0.00 a	23.38 +/– 7.46 c	0.020
Codeine	0.00 +/– 0.00 a	12.69 +/– 0.50 ab	14.31 +/– 0.66 b	0.00 +/– 0.00 a	17.47 +/– 2.10 b	0.015
Ofloxacin	0.00 +/– 0.00 a	10.13 +/– 3.32 b	13.61 +/– 2.62 b	0.00 +/– 0.00 a	18.74 +/– 5.13 b	0.021
Cotinine	1.76 +/– 0.12 a	11.71 +/– 0.11 b	11.53 +/– 0.33 b	4.68 +/– 0.07 a	6.11 +/– 0.63 ab	0.012
Erythromycin	0.06 +/– 0.03 a	4.81 +/– 0.38 b	5.97 +/– 0.39 b	0.11 +/– 0.06 a	2.10 +/– 0.30 ab	0.013
Fluoxetine	0.00 +/– 0.00 a	0.82 +/– 0.55 ac	1.47 +/– 0.39 bc	0.00 +/– 0.00 a	10.17 +/– 3.74 b	0.016
Thiabendazole	0.02 +/– 0.02	2.63 +/– 0.65	3.11 +/– 0.22	0.15 +/– 0.04	3.08 +/– 1.55	0.107
Sulfadiazine	0.43 +/– 0.01 ab	3.80 +/– 0.09 c	2.91 +/– 0.12 bc	0.03 +/– 0.03 a	0.00 +/– 0.00 a	0.010
Lincomycin	1.32 +/– 0.22	1.70 +/– 0.15	1.83 +/– 0.46	0.08 +/– 0.04	1.36 +/– 0.27	0.077
Dehydronifedipine	0.28 +/– 0.24	2.02 +/– 0.67	1.47 +/– 0.27	0.00 +/– 0.00	0.98 +/– 0.24	0.077
Sulfathiazole	0.00 +/– 0.00	0.66 +/– 0.33	0.84 +/– 0.43	0.03 +/– 0.03	0.00 +/– 0.00	0.174
Phenazone	0.00 +/– 0.00	0.15 +/– 0.15	0.33 +/– 0.05	0.00 +/– 0.00	0.00 +/– 0.00	0.052
Norgestimate	0.00 +/– 0.00	0.00 +/– 0.00	0.00 +/– 0.00	0.43 +/– 0.23	0.00 +/– 0.00	0.073
Sarafloxacin	0.00 +/– 0.00	0.00 +/– 0.00	0.34 +/– 0.34	0.05 +/– 0.05	0.00 +/– 0.00	0.519
Sulfamerazine	0.17 +/– 0.17	0.00 +/– 0.00	0.00 +/– 0.00	0.08 +/– 0.08	0.00 +/– 0.00	0.519
Miconazole	0.00 +/– 0.00	0.00 +/– 0.00	0.00 +/– 0.00	0.00 +/– 0.00	0.13 +/– 0.13	0.406
Roxithromycin	0.00 +/– 0.00	0.06 +/– 0.03	0.04 +/– 0.04	0.00 +/– 0.00	0.00 +/– 0.00	0.224
Ampicillin	0.00 +/– 0.00	0.00 +/– 0.00	0.00 +/– 0.00	0.00 +/– 0.00	0.00 +/– 0.00	NA
Cefotaxime	0.00 +/– 0.00	0.00 +/– 0.00	0.00 +/– 0.00	0.00 +/– 0.00	0.00 +/– 0.00	NA
Ciprofloxacin	0.00 +/– 0.00	0.00 +/– 0.00	0.00 +/– 0.00	0.00 +/– 0.00	0.00 +/– 0.00	NA
Clinafloxacin	0.00 +/– 0.00	0.00 +/– 0.00	0.00 +/– 0.00	0.00 +/– 0.00	0.00 +/– 0.00	NA
Digoxigenin	0.00 +/– 0.00	0.00 +/– 0.00	0.00 +/– 0.00	0.00 +/– 0.00	0.00 +/– 0.00	NA
Digoxin	0.00 +/– 0.00	0.00 +/– 0.00	0.00 +/– 0.00	0.00 +/– 0.00	0.00 +/– 0.00	NA
Enrofloxacin	0.00 +/– 0.00	0.00 +/– 0.00	0.00 +/– 0.00	0.00 +/– 0.00	0.00 +/– 0.00	NA
Flumequine	0.00 +/– 0.00	0.00 +/– 0.00	0.00 +/– 0.00	0.00 +/– 0.00	0.00 +/– 0.00	NA
Lomefloxicin	0.00 +/– 0.00	0.00 +/– 0.00	0.00 +/– 0.00	0.00 +/– 0.00	0.00 +/– 0.00	NA
Norfloxacin	0.00 +/– 0.00	0.00 +/– 0.00	0.00 +/– 0.00	0.00 +/– 0.00	0.00 +/– 0.00	NA
Ormetoprim	0.00 +/– 0.00	0.00 +/– 0.00	0.00 +/– 0.00	0.00 +/– 0.00	0.00 +/– 0.00	NA
Oxacillin	0.00 +/– 0.00	0.00 +/– 0.00	0.00 +/– 0.00	0.00 +/– 0.00	0.00 +/– 0.00	NA
Penicillin G	0.00 +/– 0.00	0.00 +/– 0.00	0.00 +/– 0.00	0.00 +/– 0.00	0.00 +/– 0.00	NA
Penicillin V	0.00 +/– 0.00	0.00 +/– 0.00	0.00 +/– 0.00	0.00 +/– 0.00	0.00 +/– 0.00	NA
Penillic Acid	0.00 +/– 0.00	0.00 +/– 0.00	0.00 +/– 0.00	0.00 +/– 0.00	0.00 +/– 0.00	NA
Sucralose	0.00 +/– 0.00	0.00 +/– 0.00	0.00 +/– 0.00	0.00 +/– 0.00	0.00 +/– 0.00	NA
Sulfachlorpyridazine	0.00 +/– 0.00	0.00 +/– 0.00	0.00 +/– 0.00	0.00 +/– 0.00	0.00 +/– 0.00	NA
Sulfamethazine	0.00 +/– 0.00	0.00 +/– 0.00	0.00 +/– 0.00	0.00 +/– 0.00	0.00 +/– 0.00	NA
Sulfamethiazole	0.00 +/– 0.00	0.00 +/– 0.00	0.00 +/– 0.00	0.00 +/– 0.00	0.00 +/– 0.00	NA

^a^Compounds are arranged in order of decreasing average concentration at the field sites.

^b^Mean values (n = 3) +/- standard error. Different letters within a row indicate significant differernces between sites based on Dunn's test.

^c^p values based on Kruskal-Wallis rank sum test.

### 3.4 Abundance of integrase gene *intI1*

**Figure 1:**
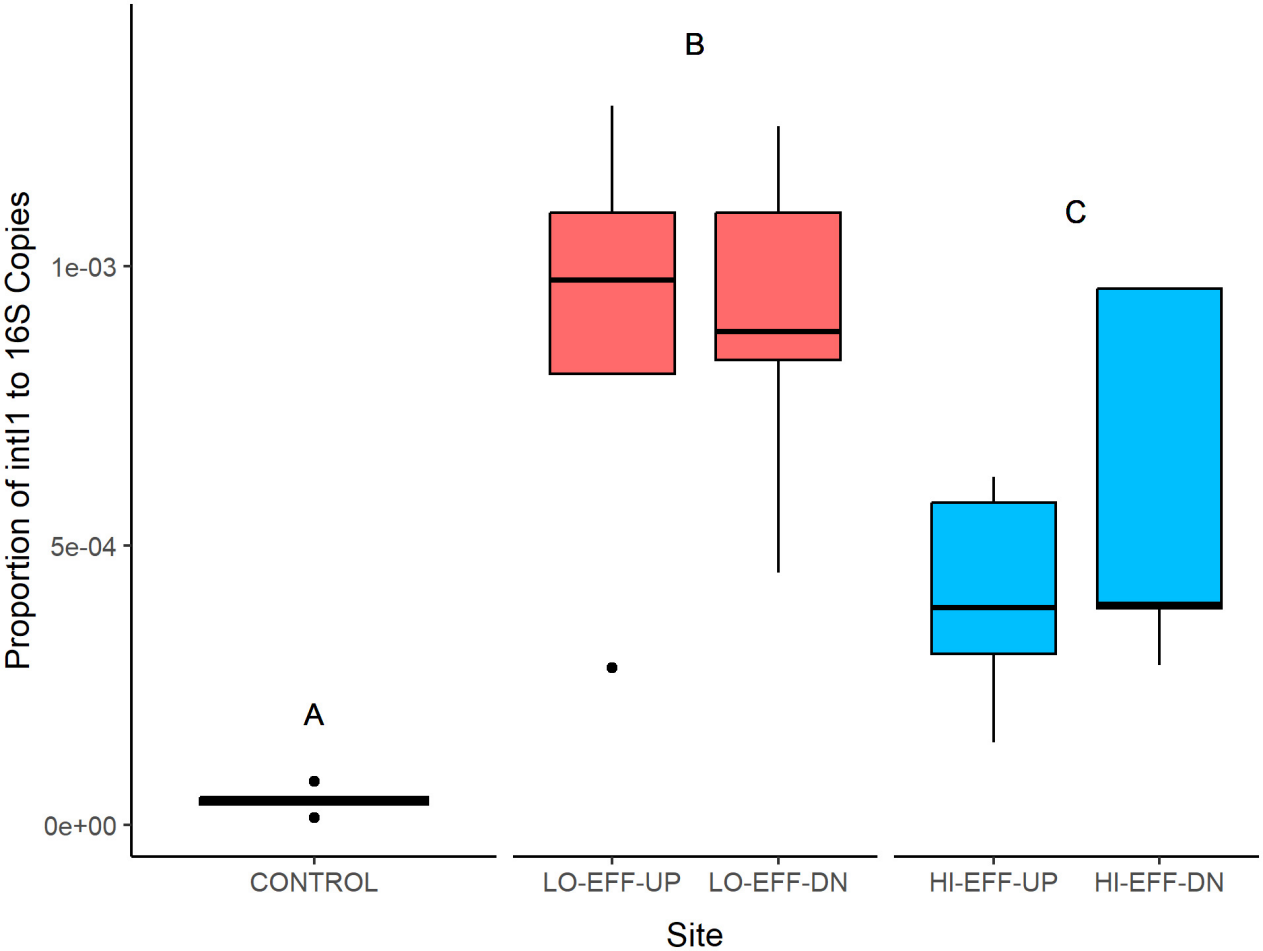
Abundance of integrase gene (intI1) in sediment samples from five field sites. Data for each site represents the median and range (*n* = 5). Different letters indicate significant differences between the sites (*p* < 0.05).

There was a significant effect of site on the abundance of *intI1* genes within the sediment samples (Figure 1). CONTROL had significantly lower *intI1* abundance than LO-EFF and HI-EFF sites (*p* < 0.0001), with HI-EFF having significantly lower *intI1* abundance than LO-EFF (p = 0.015). For both LO-EFF and HI-EFF there were no significant differences in *intI1* abundance between upstream and downstream sites.

### 3.5 Bacterial community analysis

There was a significant effect of site on the alpha diversity of the sediment bacterial communities, including both species richness (total number of observed OTUs) and Shannon diversity (Table 4), but these measures of alpha diversity did not follow the patterns observed for the stream physical and chemical characteristics or PPCP concentration reported above, i.e., there was an effect of effluent for the LO-EFF stream but not the HI-EFF stream. Specifically, both richness and diversity were significantly lower at LO-EFF-DN than at LO-EFF-UP, but there were no significant differences in richness or diversity between HI-EFF-DN and HI-EFF-UP. CONTROL had species richness and diversity values that were within the range of the values observed at the other sites.

**Table 4:** Bacterial community richness and diversity.

**Site**	**Observed species (#)^a^**	**Shannon diversity (H)^b^**
CONTROL	16,914 a	8.494 ab
LO-EFF-UP	19,641 c	8.779 c
LO-EFF-DN	15,712 d	8.212 b
HI-EFF-UP	18,637 b	8.664 ac
HI-EFF-DN	17,980 b	8.707 c
ANOVA^c^ Kruskal-Wallis^d^	*p* < 0.001^c^	*p* < 0.001^d^

^a, b^Mean values (*n* = 5) +/– standard error. Different letters within a column indicate significant differences between sites based on Tukey's test *(p* < 0.5)^a^ or Dunn's test (*p* < 0.05)^b^.

**Figure 2:**
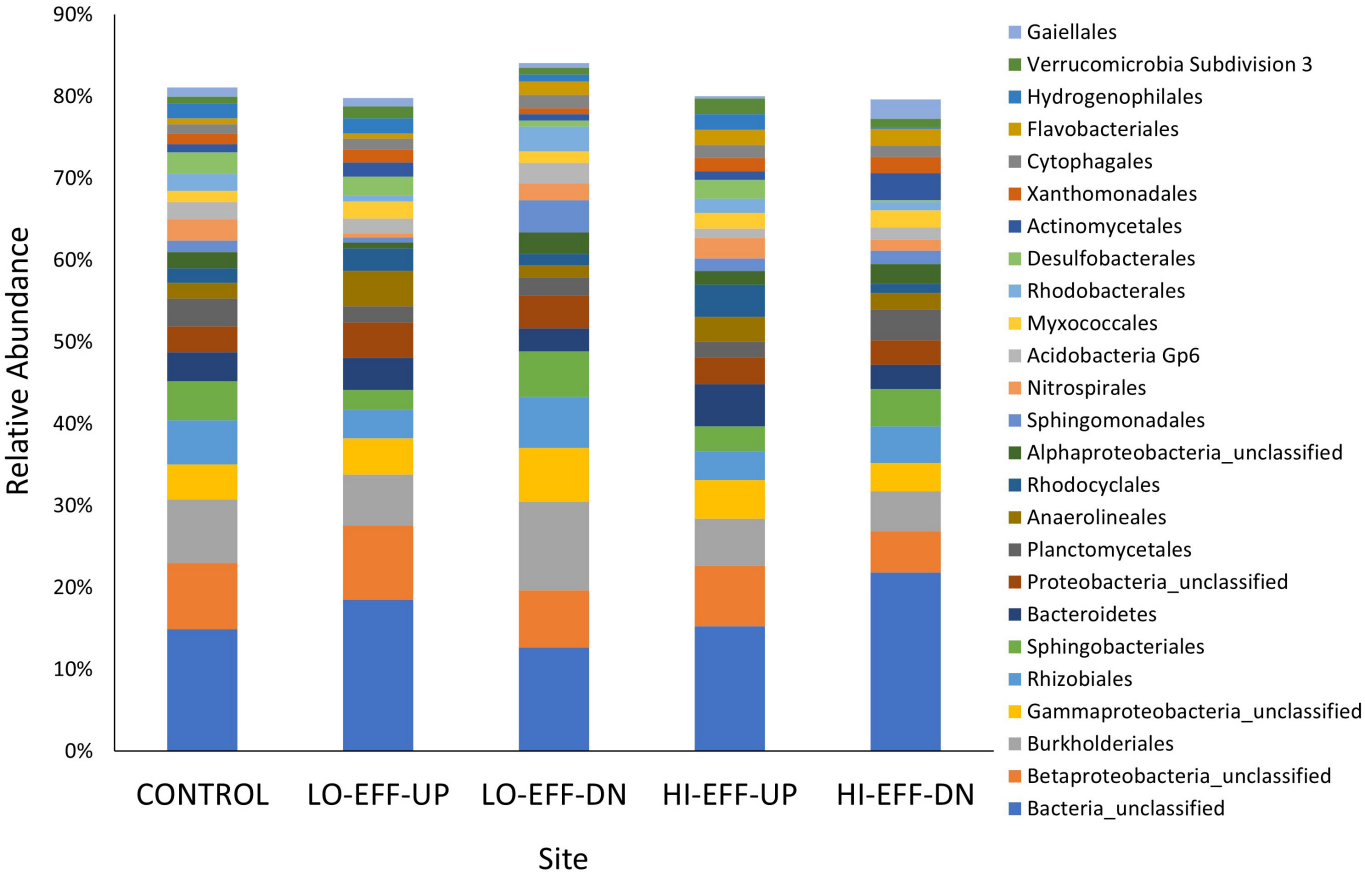
Relative abundance of the 25 most abundant bacterial orders in sediment samples from five sites. Each bar represents the mean (*n* = 5). Bacterial orders were identified based on high-throughput amplicon sequencing of partial 16 rRNA genes.

The most abundant bacterial orders identified in the sediments of all sites included an unclassified bacterial order, an unclassified betaproteobacterial order, *Burkholderiales*, an unclassified gammaproteobacterial order, *Rhizobiales*, and *Sphingobacteriales* (Figure 2). All of the sequences in the unclassified bacterial order were from a single unidentified genus, and comparison of a representative sequence from this genus to the NCBI 16S rRNA database showed the highest percent identity to multiple species within the genus *Gemmatimonas* (percent identity 88% and e value 7 x 10^−157^). There was a significant effect of site on the relative abundance of each of the 25 most abundant orders (Supplementary Table S4). Several orders varied consistently between the upstream and downstream sites of both HI-EFF and LO-EFF. For example, *Sphingobacteriales* was significantly more abundant and *Bacteroidetes, Rhodocyclales*, and *Desulfobacterales* were significantly less abundant at the downstream sites compared to the upstream sites in both streams (Supplementary Table S4). Other orders showed different trends between the two streams. For example, the unclassified bacterial order that matched to *Gemmatimonas* had opposite responses in the two streams: in HI-EFF it was significantly more abundant downstream than upstream but in LO-EFF it was significantly less abundant downstream than upstream (Supplementary Table S4). Other orders showed significant variations between downstream and upstream sites on only one stream. For example, *Rhizobiales, Sphingomonadales*, and *Rhodobacterales* were significantly more abundant at LO-EFF-DN compared to LO-EFF-UP but these orders did not vary significantly between HI-EFF-DN and HI-EFF-UP (Supplementary Table S4). Conversely, *Planctomycetales* was significantly more abundant at HI-EFF-DN compared to HI-EFF-UP but did not vary significantly between LO-EFF-DN and LO-EFF-UP (Supplementary Table S4).

**Figure 3:**
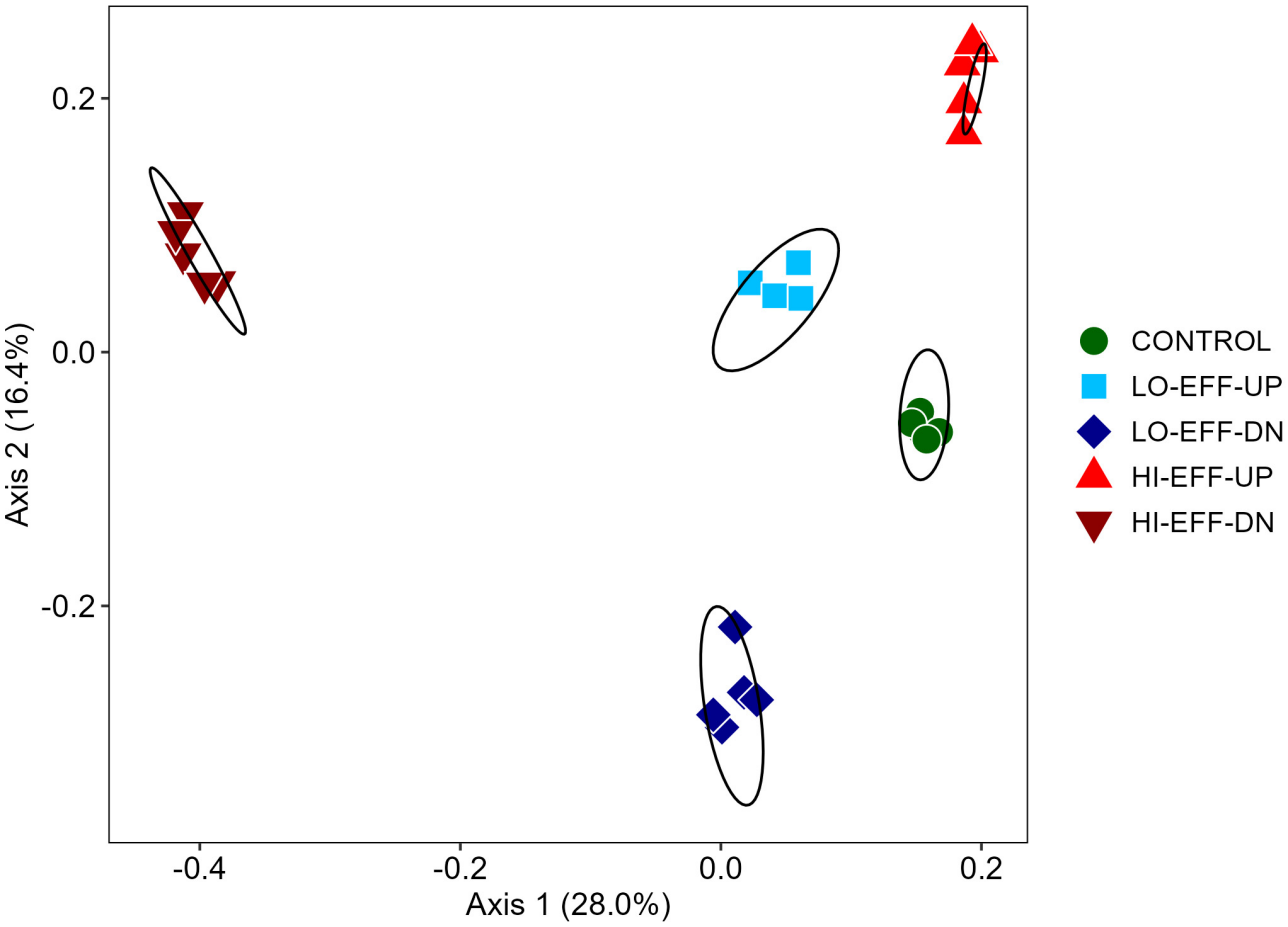
Comparison of bacterial communities from field sites based on principal coordinates analysis (PCOA) of Bray-Curtis index scores (*n* = 5). Ellipses represent 95% confidence intervals for each field site.

Comparison of the beta diversity of the sediment bacterial communities from each of the sites at the species level via principal coordinates analysis (Figure 3) and AMOVA and PERMANOVA (Supplementary Table S5) indicated significant differences in community composition between samples from each of the 5 sites. The upstream communities from both of the effluent impacted streams were relatively similar to each other and to the community from CONTROL, whereas communities from the downstream sites were highly distinct from the upstream sites and from each other (Figure 3). Metastats analysis revealed many small but statistically significant differences in species abundance between upstream and downstream sites (Tables 5, 6). A few trends were consistent at both LO-EFF and HI-EFF, including lower abundances of *Desulfuromonas, Desulfobacteraceae, Thiobacillus*, and *Sinobacteraceae* at the downstream sites, but the remainder of the changes were distinct between LO-EFF and HI-EFF, indicating that the effluent inputs were affecting the bacterial communities differently in these two streams. A direct comparison of the downstream sites from LO-EFF and HI-EFF also indicated many small but statistically significant differences in species abundance between these two wastewater-impacted sites, including lower abundances of *Comamonadaceae* and *Burkholderiales* at HI-EFF-DN (Supplementary Table S6).

**Table 5:** Bacterial OTUs with the largest differences in relative abundance between upstream and downstream sites on the high-effluent stream.

**Operation taxonomic unit^a^**	**HI-EFF-UP^b^**	**HI-EFF-DN^b^**	***p*-value^c^**
*Thiobacillus*	1.54% +/– 0.05%	0.19% +/– 0.01%	< 0.001
*Nitrospira*	1.29% +/– 0.13%	0.24% +/– 0.07%	< 0.001
*Bacteria*	0.01% +/– 0.00%	0.63% +/– 0.02%	< 0.001
*Comamonadaceae*	1.14% +/– 0.05%	0.54% +/– 0.03%	< 0.001
*Mycobacterium*	0.04% +/– 0.01%	0.62% +/– 0.09%	< 0.001
*Bacteria_unclassified*	0.00% +/– 0.00%	0.57% +/– 0.03%	< 0.001
*Betaproteobacteria*	0.57% +/– 0.04%	0.01% +/– 0.00%	< 0.001
*Rhodobacteraceae*	1.01% +/– 0.03%	0.47% +/– 0.03%	< 0.001
*Nitrospira*	0.64% +/– 0.02%	0.10% +/– 0.03%	< 0.001
*Nitrospira*	0.25% +/– 0.02%	0.77% +/– 0.09%	0.001
*Dechloromonas*	0.61% +/– 0.01%	0.09% +/– 0.01%	< 0.001
*Sinobacteraceae*	0.83% +/– 0.09%	0.32% +/– 0.03%	0.001
*Rhodocyclaceae*	0.60% +/– 0.05%	0.10% +/– 0.01%	< 0.001
*Planctomycetaceae*	0.00% +/– 0.00%	0.47% +/– 0.07%	< 0.001
*Actinomycetales*	0.00% +/– 0.00%	0.47% +/– 0.05%	< 0.001
*Desulfuromonas*	0.47% +/– 0.04%	0.03% +/– 0.00%	< 0.001
*Gaiella*	0.03% +/– 0.00%	0.46% +/– 0.07%	0.001
*Proteobacteria*	0.46% +/– 0.07%	0.03% +/– 0.01%	0.001
*Desulfobacteraceae*	0.45% +/– 0.03%	0.02% +/– 0.01%	< 0.001
*Bacteroidetes*	0.45% +/– 0.01%	0.04% +/– 0.00%	< 0.001

^a^OTUs were assigned to the lowest possible taxonomic rank and are listed in order of decreasing difference in relative abundance between upstream and downstream sites.

^b^Mean values +/- standard error *(n* = 5).

^c^Based on one way ANOVA.

**Table 6:** Bacterial OTUs with the largest differences in relative abundance between upstream and downstream sites on the low-effluent stream.

**Operation taxonomic unit^a^**	**LO–EFF–UP^b^**	**LO–EFF–DN^b^**	***p*–value^c^**
*Sphingorhabdus*	0.11% +/– 0.01%	1.29% +/– 0.13%	< 0.001
*Rhodobacteraceae*	0.32% +/– 0.02%	1.35% +/– 0.13%	< 0.001
*Comamonadaceae*	0.83% +/– 0.13%	1.77% +/– 0.21%	0.003
*Gammaproteobacteria*	0.06% +/– 0.00%	0.96% +/– 0.16%	0.001
*Rhodobacteraceae*	0.12% +/– 0.01%	1.00% +/– 0.13%	< 0.001
*Nitrospira*	0.21% +/– 0.03%	0.98% +/– 0.14%	0.001
*Thiobacillus*	1.44% +/– 0.10%	0.67% +/– 0.08%	< 0.001
*Sinobacteraceae*	0.93% +/– 0.05%	0.20% +/– 0.02%	< 0.001
*Sphingorhabdus*	0.01% +/– 0.00%	0.72% +/– 0.10%	< 0.001
*Burkholderiales*	0.56% +/– 0.07%	1.17% +/– 0.15%	0.004
*Burkholderiales*	0.84% +/– 0.08%	1.44% +/– 0.11%	0.002
*Novosphingobium*	0.15% +/– 0.01%	0.75% +/– 0.06%	< 0.001
*Hydrogenophaga*	0.10% +/– 0.01%	0.67% +/– 0.09%	< 0.001
*Betaproteobacteria*	0.86% +/– 0.07%	0.33% +/– 0.03%	< 0.001
*Sulfurisoma*	0.75% +/– 0.06%	0.22% +/– 0.03%	< 0.001
*Nitrospira*	0.10% +/– 0.02%	0.61% +/– 0.13%	0.003
*Comamonadaceae*	1.29% +/– 0.10%	1.76% +/– 0.12%	0.012
*Bacteroidetes*	0.59% +/– 0.02%	0.13% +/– 0.01%	< 0.001
*Rhizobiales*	0.10% +/– 0.01%	0.52% +/– 0.05%	< 0.001
*Bacteria*	0.48% +/– 0.01%	0.08% +/– 0.01%	< 0.001
*Desulfuromonas*	0.37% +/– 0.04%	0.07% +/– 0.01%	< 0.001
*Desulfobacteraceae*	0.42% +/– 0.03%	0.17% +/– 0.03%	< 0.001

^a^OTUs were assigned to the lowest possible taxonomic rank and are listed in order of decreasing difference in relative abundance between upstream and downstream sites.

^b^Mean values +/– standard error (*n* = 5).

^c^Based on one way ANOVA.

## 4 Discussion

There were multiple differences in the physical and chemical properties of the upstream and downstream sites on the stream that received a higher percentage of its flow (~80%) from WWTP effluent (HI-EFF), as we had predicted. The observed changes included increased water column concentrations of nitrate and SRP, increased water temperature, and decreased sediment organic matter at the downstream site compared to the upstream site. Similar effects of WWTP effluent have been reported previously (Chambers and Prepas, 1994; Marti et al., 2004; Gücker et al., 2006; Spänhoff et al., 2007; Waiser et al., 2011a; Drury et al., 2013a), indicating that these may be common impacts of effluent inputs. We had hypothesized that the physical and chemical changes resulting from the high effluent input would impact the stream microbial communities, and we observed lower concentrations of phycocyanin and chlorophyll-a in the water column at HI-EFF-DN compared to HI-EFF-UP. Phycocyanin is an accessory pigment that is found in cyanobacteria and chlorophyll-a is a photosynthetic pigment found in algae and cyanobacteria, and these pigments are commonly used as indicators of the abundance of these organisms in aquatic habitats (Pasztaleniec et al., 2020). The decreased abundance of photosynthetic microorganisms at HI-EFF-DN is ecologically important because these organisms are key drivers of primary production in stream ecosystems and represent important food resources for higher trophic levels. The fact that photosynthetic microorganisms were less abundant at HI-EFF-DN was surprising because the higher concentrations of inorganic nitrogen and phosphorous as well as the increased water temperature and decreased turbidity would all be expected to increase the abundance of these organisms, suggesting that some other aspects of the effluent were negatively impacting them, such as PPCPs or other pollutants. Previous studies using experimental stream mesocosms have demonstrated the potential for PPCP exposure to reduce chlorophyll-a concentration (Lee et al., 2016; Rosi-Marshall et al., 2013) and algal abundance (Drury et al., 2013b; Corcoll et al., 2015). Furthermore, a study at several sites in France indicated that WWTP effluent increased PPCP concentrations and reduced cyanobacterial abundance (Aubertheau et al., 2017).

Concentrations of 10 PPCPs were significantly higher at HI-EFF-DN compared to HI-EFF-UP, indicating that high effluent input significantly increased the concentrations of PPCPs at this site, as we had predicted. The PPCPs that were elevated at HI-EFF-DN included several antibiotics (sulfamethoxazole, sulfanilamide, clarithromycin, azithromycin, and ofloxacin), which might be expected to negatively impact cyanobacteria, which are prokaryotes, and thus could have contributed to the decreased concentrations of phycocyanin and chlorophyll-a at HI-EFF-DN. The antihistamine diphenhydramine and the antidepressant fluoxetine were also significantly higher at HI-EFF-DN compared to HI-EFF-UP, and previous reports indicated that experimental exposure to these specific compounds significantly reduced photosynthesis rates (gross primary production) in stream biofilms (Rosi-Marshall et al., 2013; Richmond et al., 2016). Therefore, the elevated concentrations of diphenhydramine and fluoxetine at HI-EFF-DN could also have contributed to the decreased abundance of photosynthetic organisms.

In contrast to the HI-EFF stream, there were no significant differences in the physical and chemical properties or PPCP concentrations of the upstream and downstream sites of the stream (LO-EFF) that received a smaller percentage of its flow (~13%) from WWTP effluent, which also matched our prediction. The similar physical and chemical properties of LO-EFF-DN and LO-EFF-UP corresponded with a similar abundance of photosynthetic microorganisms in the water column at these sites, based on the cyanobacterial and algal indicators phycocyanin and chlorophyll-a. However, there was a significant difference in the diversity of sediment bacterial communities in the LO-EFF stream, with LO-EFF-DN having significantly lower species richness and Shannon diversity than LO-EFF-UP. This was surprising because none of the measured physical and chemical characteristics varied significantly between these sites. A previous study by our group conducted on two other Illinois rivers also showed significant decreases in species richness and diversity in sediment bacterial communities downstream of WWTP effluent inputs (Drury et al., 2013a), suggesting that this may be a generalizable effect of effluent addition, although we did not observe this change in the HI-EFF stream in the current study. These conflicting results for the LO-EFF and HI-EFF streams may be site specific or may be based on differences in the contribution of effluent to stream flow, e.g., low effluent inputs might decrease diversity and high inputs might enhance diversity. Additional work at a broader range of sites will be needed to fully assess the effects of effluent on bacterial community diversity.

In both the effluent-receiving streams there were significant differences in the composition of sediment bacterial communities downstream of the effluent input points as compared to upstream, a trend which has been reported previously (Wakelin et al., 2008; Drury et al., 2013a; Hagberg et al., 2021). PCOA analysis indicated that bacterial communities at both of the upstream sites were more similar to the CONTROL site than to their respective downstream sites, and that effluent input changed the bacterial communities in the two effluent-receiving streams along distinct trajectories. Four notable similarities emerged, with the downstream sites on both effluent-receiving streams showing significantly higher abundance of bacteria from the order *Sphingobacteriales* and significantly lower abundance of bacteria from the orders *Bacteroidetes, Rhodocyclales*, and *Desulfobacterales*.

The order *Sphingobacteriales* was significantly higher in abundance at the downstream sites compared to the upstream sites of both effluent-receiving streams. *Sphingobacteriales* are an order of Gram-negative bacteria found in a wide range of ecosystems that are known for their ability to metabolize anthropogenic compounds, including herbicides and antimicrobials (Kämpfer, 2010). *Sphingobacteriales* have previously been documented to increase in abundance downstream of WWTP effluent inputs (Drury et al., 2013a) and in response to increased PPCP concentrations (Hagberg et al., 2021). A previous study by our group indicated that experimental exposure of stream bacterial communities to the antibiotic ciprofloxacin resulted in increased abundance of a *Pedobacter* (Rosi et al., 2018), a genus from the order *Sphingobacteriales*, further supporting the hypothesis that PPCPs may select for bacteria from this order. Selection by PPCPs could explain the elevated abundance of *Sphingobacteriales* at HI-EFF-DN, which had significantly higher concentrations of multiple PPCPs. However, *Sphingobacteriales* were also elevated at LO-EFF-DN, where we did not detect significantly higher concentrations of any PPCPs or any changes in stream physical and chemical characteristics. It is possible that the increased abundance of *Sphingobacteriales* at LO-EFF-DN might have been linked to anthropogenic compounds that were not assayed for in this study, or to some other impact of WWTP that was not measured. Interestingly, the abundance of *Sphingobacteriales* at the CONTROL site was elevated compared to the upstream sites and was equivalent to the downstream sites on both effluent-receiving streams. This may reflect the high level of agriculture in the watershed of the CONTROL stream, which could have contributed some anthropogenic compounds not measured in this study, e.g., herbicides.

The bacterial order *Bacteroidetes* was significantly less abundant at the downstream sites compared to upstream sites on both effluent-receiving streams, and the abundance of this order in the CONTROL stream was intermediate between the upstream and downstream sites. *Bacteroidetes* are an order of Gram-negative bacteria that are widely distributed in nature and display a wide range of metabolisms (Parte et al., 2011). All of the sequences assigned to this order in our study were from a single unidentified genus, and BLAST comparison of a representative sequence from this genus to the NCBI 16S rRNA database showed the highest percent identity to multiple species within the genus *Flavobacterium* (percent identity 99% and e value 5 × 10^−157^). *Flavobacterium* are widely distributed in nature, occurring mostly in aquatic ecosystems (Bernardet and Bowman, 2006) where they are involved in the metabolism of various plant associated organic compounds, including carbohydrates, polysaccharides, cellulose derivatives (Bernardet and Bowman, 2006) and cellulose (Lednická et al., 2000). Our data suggest a negative impact of WWTP effluent on the relative abundance of *Flavobacterium*, which could have negative implications for organic matter breakdown and nutrient cycling in these stream ecosystems. Several previous studies indicated that experimental exposure of stream bacterial communities to PPCPs resulted in decreased relative abundance of *Flavobacterium* (Rosi et al., 2018; Rosi-Marshall et al., 2013), suggesting that the decrease observed in the current study might be linked to these pollutants. Specifically, diphenhydramine exposure resulted in significant decreases in *Flavobacterium* (Rosi-Marshall et al., 2013), and diphenhydramine was elevated at HI-EFF-DN.

The bacterial order *Rhodocyclales* was also significantly less abundant at the downstream sites compared to upstream sites on both effluent-receiving streams, and the abundance of this order in the CONTROL stream was intermediate between the upstream and downstream sites. *Rhodocyclales* is an order of Gram-negative bacteria that includes taxa with a wide range of metabolic lifestyles (Oren, 2014). The majority (60%) of the sequences assigned to this order in our study were from a single unidentified genus, and BLAST comparison of a representative sequence from this genus to the NCBI 16S rRNA database showed that it was most closely related to *Dechloromonas denitrificans* (percent identity 98% and e value 4 x 10^−146^). Metastats analysis indicated that another OTU assigned to the genus *Dechloromonas* was also significantly lower in abundance at HI-EFF-DN compared to HI-EFF-UP. Bacteria from the genus *Dechloromonas*, and the species *Dechloromonas denitrificans* in particular, conduct denitrification (Oren, 2014), an important step in the nitrogen cycle that can remove nitrogen from aquatic ecosystems and limit nutrient pollution and eutrophication, so a negative effect of WWTP effluent on this taxon would be ecologically significant.

Another common trend observed on both effluent-receiving streams was a significant decrease in sulfur reducing bacteria at the downstream sites. Specifically, the bacterial order *Desulfobacterales* was significantly less abundant at the downstream sites compared to upstream sites, and one species from this order, identified as *Desulfobacteraceae*, was significantly less abundant at the downstream sites compared to upstream sites on both streams. Furthermore, the OTU *Desulfuromonas*, another sulfur reducing taxon, was also less abundant at the downstream sites compared to upstream sites on both streams. A previous study reported a similar trend, with one sulfate-reducing genus from the *Desulfobacterales* order, *Desulfococcus*, being significantly less abundant in two streams downstream of WWTP effluent inputs (Drury et al., 2013a), suggesting that decreased abundance of sulfur reducers may be a common result of effluent inputs. The potential negative effect of effluent on sulfur reducers is further supported by the fact that the decrease in *Desulfobacteraceae* was more pronounced in the high effluent stream than the low effluent stream, and the fact that the abundance of the *Desulfobacterales* order at the CONTROL site was equivalent to the upstream sites and significantly higher than the downstream sites on both HI-EFF and LO-EFF. Sulfur reduction is an ecologically important process that contributes to organic matter breakdown and carbon cycling in anoxic habitats, which can be found in stream sediments, so a negative effect of effluent on these organisms is ecologically significant. Two potential drivers of the decreases in sulfur reducers, which are strict anaerobes (Thauer et al., 2007), could be the higher dissolved oxygen and nitrate concentrations at the downstream sites, which would offer more energetically favorable electron acceptors than sulfate.

Despite the similarities described above, PCOA indicated some significant differences between the wastewater-impacted downstream sites from the two streams, and several differences were apparent at the order level. For example, an unclassified bacterial order and *Actinomycetales* were significantly more abundant at HI-EFF-DN compared to LO-EFF-DN and *Burkholderiales*, a Gammaproteobacterial order, and *Rhodobacterales* were significantly less abundant at HI-EFF-DN compared LO-EFF-DN. For the unclassified bacterial order that was significantly more abundant at HI-EFF-DN compared LO-EFF-DN, all of the sequences were from a single genus that we identified as *Gemmatimonas*. *Gemmatimonas* are Gram-negative, heterotrophic organisms that grow under aerobic conditions (Parte et al., 2011). *Gemmatimonas* are one of the most common soil bacterial taxa and are also found in freshwater and marine environments, and they have also been identified as abundant within wastewater treatment systems (Mujakić et al., 2022), so their high abundance at HI-EFF-DN may reflect the major contribution of effluent to this site. *Gemmatimonas* species have also been shown to be naturally resistant to some antibiotics (Mujakić et al., 2022), so the elevated concentration of PPCPs at HI-EFF-DN may also have contributed to the high abundance of this organism.

*Actinomycetales* abundance was more than 3 times higher at HI-EFF-DN compared to LO-EFF-DN. *Actinomycetales* are a diverse group of Gram-positive bacteria that are ubiquitous in soil and aquatic habitats and are associated with decomposition of organic material (Parte et al., 2012). Many species of *Actinomycetales* produce antimicrobial compounds (Takahashi and Nakashima, 2018) and possess natural resistance mechanisms to the antibiotics they produce (Sugiyama, 2015). Two of the antibiotics that were elevated at HI-EFF-DN, clarithromycin and azithromycin, are semi-synthetic antibiotics derived from erythromycin, which is produced by actinomycetes. Therefore, it is possible that the high abundance of *Actinomycetales* at HI-EFF-DN might be linked to their resistance to the high concentration of antibiotics at this site.

*Burkholderiales* were significantly less abundant at HI-EFF-DN compared to LO-EFF-DN. Within this order 33% of the sequences were assigned to the genus *Comamonadaceae*. Metastats analysis also indicated that two *Comamonadaceae* species were significantly less abundant at HI-EFF-DN as compared to LO-EFF-DN. *Comamonadaceae* are Gram-negative aerobic heterotrophs that are common in both terrestrial and aquatic habitats and are known for their ability to degrade a wide range of complex organic compounds (Garrity et al., 2005). A previous study by our group indicated that experimental exposure of stream bacterial communities to the antibiotic ciprofloxacin resulted in decreased relative abundance of *Comamonadaceae* (Rosi et al., 2018), suggesting that the high PPCP concentration may have selected against *Comamonadaceae*.

*Rhodobacterales* were significantly less abundant at HI-EFF-DN compared to LO-EFF-DN. Almost all (94%) of the sequences assigned to this order in our study were from a single unidentified genus, and comparison of a representative sequence from this unidentified genus to the NCBI 16S rRNA database showed the highest percent identity to multiple species within the genus *Rhodobacter* (percent identity 99% and e value 3 × 10^−147^). *Rhodobacter* are a physiologically diverse group of generally aquatic purple non-sulfur bacteria and are capable of a wide range of metabolisms, including photoheterotrophy and photoautotrophy (Garrity et al., 2005). Work at a small stream in Sweden reported decreased abundance of *Rhodobacter* downstream of a WWTP and suggested that the decrease might have been driven by the increased concentration of PPCPs that was also observed at the site (Hagberg et al., 2021). Although *Rhodobacter* does not contain phycocyanin or chlorophyll-a, the low abundance of this phototrophic organism at HI-EFF-DN parallels the low concentration of those photosynthetic pigments observed at that site and suggests a generally negative effect of WWTP associated PPCPs on phototrophic microbes.

In addition to the above comparisons between the upstream and downstream sites on the two urban, effluent-receiving streams, which we have used to assess the effects of WTTP effluent, comparisons of the rural control stream and the two urban streams enabled assessment of watershed-scale trends. The watersheds of the control and urban streams differed dramatically in land use, with the dominant land use categories for both of the effluent-receiving streams being residential, commercial, and transportation, and the dominant land use category for the control stream being agriculture. The control site had the lowest concentrations of the PPCPs detected in this study, with multiple PPCPs that were detected in the urban streams not detected in the control stream, and several PPCPs had higher concentrations in the upstream sites of the urban streams than the control stream (e.g., caffeine, 1,7-dimethylxanthine, and acetaminophen), suggesting that in addition to WWTP effluent, non-point sources in the more urbanized watersheds may be contributing PPCPs to those streams. The urban streams, at both the upstream and downstream sites, also had lower abundances of photosynthetic pigments compared to the control stream, suggesting a negative impact of PPCPs or other watershed-scale characteristics on photosynthetic microorganisms. Finally, the urban streams, at both the upstream and downstream locations, had significantly higher abundances of the clinical class 1 integrase gene, *intI1*, which is an indicator of bacterial community resistance to a variety of anthropogenic stressors, including PPCPs. This result suggests that the elevated concentrations of PPCPs in the urban streams, originating from both effluent and non-point sources, may be resulting in higher abundance of this gene, which agrees with our prediction. It was interesting that there was no significant difference in *intI1* abundance between the upstream and downstream sites in the HI-EFF stream despite the fact that there were 10 PPCPs that had significantly higher concentrations at HI-EFF-DN compared to HI-EFF-UP. However, several PPCPs had concentrations at HI-EFF-UP that were higher or equivalent to HI-EFF-DN, including caffeine, acetaminophen, and cotinine, which may explain the lack of difference in *intI1* abundance between these sites. It was also interesting that *intI1* abundance was higher in LO-EFF than HI-EFF. This suggests that some site-specific characteristics were the key drivers of *intI1* abundance rather than effluent input. Drivers of *intI1* abundance could include concentrations of some specific PPCPs or combinations of PPCPs, e.g., acetaminophen and 1,7-dimethylxanthine were higher in LO-EFF than HI-EFF, including both upstream and downstream sites. Drivers of *intI1* abundance could also include watershed land use, e.g., LO-EFF had higher proportions of commercial and industrial land use, which could contribute anthropogenic contaminants not measured in this study. A more complete assessment of the drivers of *intI1* abundance in stream microbial communities will require additional work at a broader range of sites.

Some limitations of the current study should be acknowledged. First, the field sampling was limited to three streams, so it is not possible to definitively separate effluent effects from site effects. For example, the decreases in bacterial species richness and diversity observed at the low effluent downstream site may not be linked to effluent inputs since the same trends were not observed for the high effluent stream. Therefore, care should be taken in extrapolating results of this study to other sites. However, as discussed above, many of the specific downstream changes observed for the high effluent stream have been observed in other studies of effluent impacts or studies of pharmaceutical impacts, adding confidence to interpreting these changes as effluent effects. Second, the relatively short distance between sediment replicates at each site (~1 m) limits their biological independence, although previous work has demonstrated a significant level of spatial variation in sediment bacterial communities over limited distances in streams, especially at sites downstream of WWTPs (Drury et al., 2013a,b; Hoellein et al., 2017). Third, sediment samples were stored overnight at 4°C before storage at −20°C and subsequent DNA extraction. While storage at 4°C should limit microbial activity and changes in community composition, some changes could have occurred during storage. Finally, *intI1* copy numbers were normalized to 16S rRNA gene copy numbers as a proxy for the size of the bacterial communities in the samples, which is a standard approach for quantification of *intI1* (e.g., see Gillings et al., 2015) as well as other functional genes (Smith and Osborn, 2009). However, 16S rRNA gene copy number varies widely between bacterial taxa, from one to up to 15 copies per genome (Klappenbach et al., 2001), so it is an imperfect proxy for the size of the bacterial communities and can confound comparisons of *intI1* copy numbers between communities with different taxonomic compositions.

## Data Availability

The original contributions presented in the study are publicly available. This data can be found in here: https://www.ncbi.nlm.nih.gov/, accession number PRJNA66291.
