# Supplementary material for: Treated wastewater effluent increases pharmaceutical concentrations and alters benthic microbial communities in streams
Source: Front Microbiol. 2025 Aug 26;16:1649739. doi: 10.3389/fmicb.2025.1649739 (PMC12417413; doi:10.3389/fmicb.2025.1649739)
Supplement: Supplementary file 1 [file Data_Sheet_1.pdf]

Treated Wastewater Effluent Increases Pharmaceutical Concentrations and Alters Benthic Microbial Communities in Streams

Benjamin Lorentz[1], Maddee Rauhauser[2], Ryan Krantz[1], Daniel Snow[2], John J. Kelly[1,*]
[1]Loyola University Chicago, Chicago, IL
[2]University of Nebraska, Lincoln, NE

Supplemental Material

*** Correspondence:**

John Kelly

Department of Biology

Loyola University Chicago

1032 West Sheridan Rd.

Chicago, IL 60660

phone: 773.508.3681

email: jkelly7@luc.edu

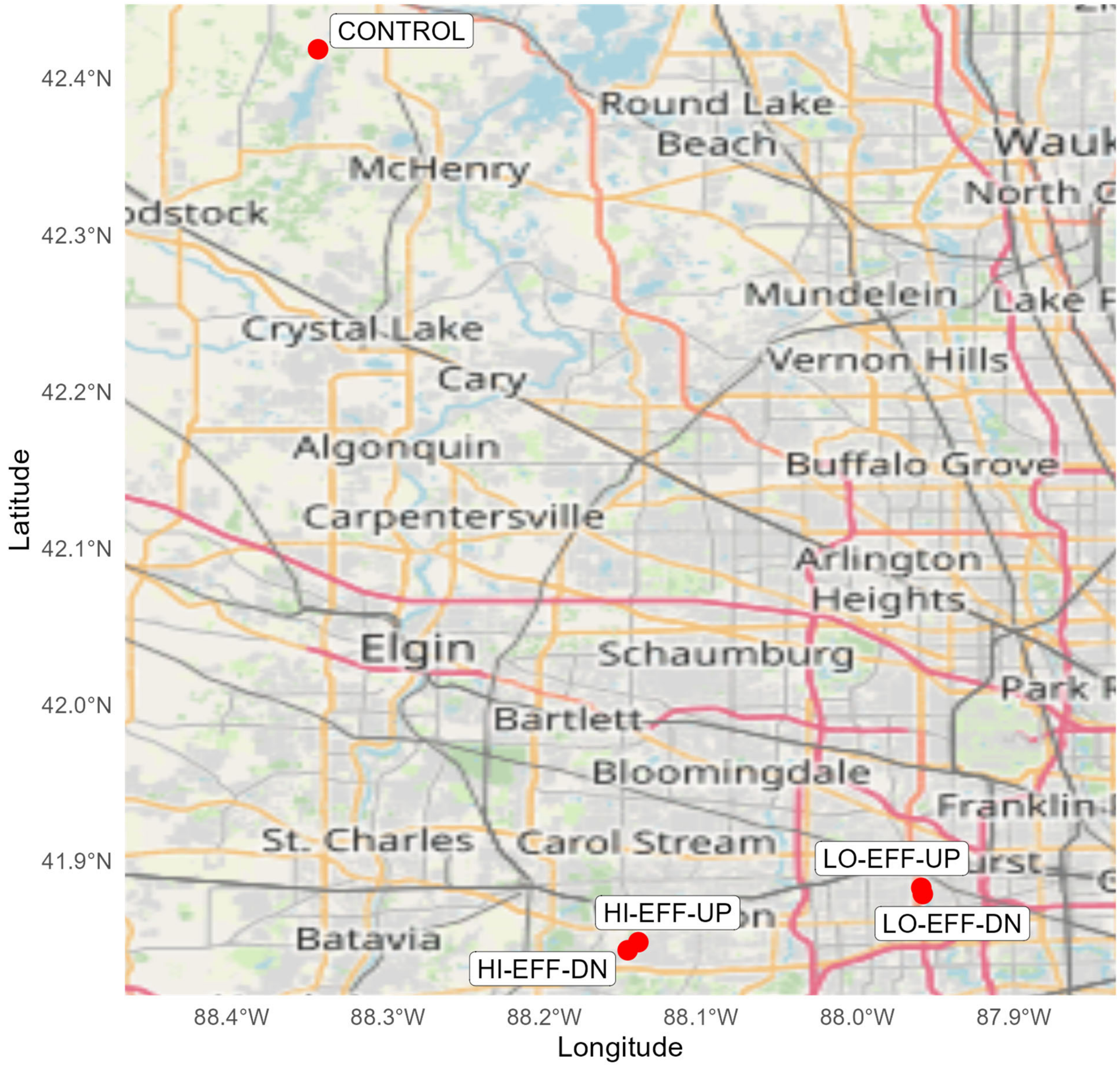

Figure S1. Map of field locations

Table S1. Uni-Spray source and instrumental conditions

Impactor Voltage (kV)	1.5
Source Temperature (°C)	150
Desolvation Temp (°C)	600
Desolvation Gas Flow (L/Hr)	1000
Cone Gas Flow (L/Hr)	1
LM Resolution 1	4.7
HM Resolution 1	15.1
Ion Energy 1	-0.5
Entrance	1
Exit	1
LM Resolution 2	7.6
HM Resolution 2	15.2
Ion Energy 2	0.5
Ion Guide 2 Offset (V)	0.5
Detector Gain	1

Table S2. MRM transitions, cone voltage, collision energies, and retention times.

Compound	Parent-Daughter m/z	Cone (V)	Collision (V)	Retention Time (min)
Acetaminophen-(acetyl-13C2,15N) (IS)	155.108>92.949<br>155.108>44.939	36	26<br>24	2.17
Ciprofloxacin-(carboxyl-13C3, quinoline-15N) (IS)	336.068>318<br>336.068>248.105	54	20<br>24	2.76
Caffeine-(trimethyl-13C3) (IS)	198.074>42.926<br>198.074>140.034	46	46<br>28	2.58
Trimetroprim-(pyrimidine-13C3) (IS)	294.14>126.042<br>294.14>264.043	64	30<br>32	2.67
Carbamazepine-13C6 (IS)	243.11>171.049<br>243.11>184.905	42	50<br>44	4.91
Sulfamethazine-(phenyl-13C6) (IS)	285.067>97.953<br>285.067>123.971	44	44<br>34	2.73
Sulfamethoxazole-13C6 (IS)	260.134>98.071<br>260.134>162	16	38<br>20	3.00
Thiabendazole-13C6 (IS)	208.062>180.982<br>208.062>137.003	54	22<br>32	2.66
Erythromycin-(N-methyl-13C, D3) (IS)	738.398>82.986<br>738.398>162.130	16	70<br>48	5.07
Cotinine-(methyl-d3) (IS)	180.134>80<br>180.134>100.949	52	32<br>30	1.14
Fluoxetine-d6 (IS)	316.170>43.934<br>316.170>154.134	34	20<br>10	5.33
Atrazine-13C3 (Sur)	219.117>97.966<br>219.117>105.903	46	32<br>40	5.00
Des-ethyl atrazine-13C3 (Sur)	191>149<br>191>82	30	20<br>20	3.07
Orphenadrine (Sur)	270.093>181.129<br>270.093>165.258	12	16<br>62	5.03
Oleandomycin (Sur)	688.381>158.159<br>688.381>544.271	42	30<br>18	4.86
1,7-Dimethylxanthine	181.2>124<br>181.2>96	40	25<br>35	2.39
Acetaminophen	152.064>92.828<br>152.064>64.979	50	22<br>28	2.17
Ampicillin	351.846>265.112<br>351.846>43.964	60	24<br>38	2.81
Azithromycin	749.438>158.159<br>749.438>83.048	62	38<br>60	5.44
Caffeine	195.094>138.027<br>195.094>41.948	60	18<br>34	2.58
Carbamazepine	237.11>178.953<br>237.11>165.094	36	34<br>38	4.91

Ciprofloxacin	332.117>314<br>332.117>231.076	54	15<br>36	2.76
Clarithromycin	748.344>158.099<br>748.344>83.049	44	28<br>50	5.44
Codeine	300.137>58.014<br>300.137>215.084	68	30<br>24	2.47
Cotinine	177.124>80.014<br>177.124>97.973	40	20<br>20	1.15
Danofloxacin	358.157>340<br>358.157>82.073	58	15<br>48	2.81
Dehydronifedipine	345.077>284.098<br>345.077>267.972	64	26<br>26	5.22
Digoxigenin	391.159>355.171<br>391.159>337.218	46	10<br>14	3.29
Digoxin	781.344>97.029<br>781.344>651.297	74	28<br>10	5.19
Diltiazem	415.064>178.006<br>415.064>108.938	56	24<br>60	4.98
Diphenhydramine	256.19>167.024<br>256.19>152.001	34	14<br>48	4.69
Enrofloxacin	360.3>342.3<br>360.3>316.3	56	20<br>20	2.85
Erythromycin	734.334>158.094<br>734.334>83.047	54	28<br>56	5.07
Flumequine	262.09>202.003<br>262.09>126.009	30	30<br>48	4.84
Fluoxetine	310.17>43.963<br>310.17>148.103	38	10<br>6	5.34
Lincomycin	407.189>126.104<br>407.189>41.821	46	28<br>70	2.59
Lomefloxacin	352.127>265.081<br>352.127>43.962	68	22<br>30	2.82
Miconazole	416.97>158.505<br>416.97>161.426	80	28<br>26	6.07
Norfloxacin	320.107>302<br>320.107>233.106	62	15<br>22	2.74
Norgestimate	370.149>124.029<br>370.149>91.052	84	30<br>46	6.30
Ofloxacin	362.083>318<br>362.083>261.056	66	15<br>26	2.73
Ormetoprim	275.108>123.054<br>275.108>81.037	62	24<br>40	2.76
Oxacillin (MeOH)	434.068>160.006<br>434.068>144.007	46	14<br>32	5.07
Penicillin G (MeOH)	367.1319>160.012<br>367.1319>91.074	2	14<br>56	4.58

Penicillin V (MeOH)	383.004>160.015<br>383.004>114.033	36	14<br>38	4.96
Phenazone	189.132>55.999<br>189.132>77.014	50	28<br>36	2.85
Roxithromycin	837.38>158.096<br>837.38>116.021	62	38<br>46	5.48
Sarafloxacin	386.137>299.035<br>386.137>348.068	52	24<br>30	3.01
Sucralose (Na)	418.904>220.993<br>418.904>238.876	52	18<br>18	2.82
Sulfachloropyridazine	285.1>156<br>285.1>92	32	15<br>28	2.91
Sulfadiazine	251.057>91.967<br>251.057>155.968	44	26<br>12	2.32
Sulfadimethoxine	311.107>91.962<br>311.107>156.023	58	32<br>18	3.68
Sulfamerazine	265.087>91.964<br>265.087>155.962	50	26<br>16	2.56
Sulfamethazine	279.107>91.973<br>279.107>186.033	36	30<br>16	2.73
Sulfamethizole	271.043>91.973<br>271.043>155.981	38	28<br>12	2.74
Sulfamethoxazole	254.057>92.032<br>254.057>155.973	14	24<br>14	3.00
Sulfanilamide	173.104>92<br>173.104>156	38	20<br>10	0.61
Sulfathiazole	255.969>155.987<br>255.969>91.975	34	14<br>26	2.47
Thiabendazole	202.026>175.017<br>202.026>131.052	36	22<br>30	2.66
Trimethoprim	291.098>123.053<br>291.098>230.041	68	22<br>22	2.67

Table S3. Instrument detection limits, and method detection limits of analytes measured in cartridge extracts.

Compound	IDL (pg)	Spiked at 2 and 20 ng/g MDL (ng/g)	AVG REC (%)
Atrazine-13C3 (Sur)	0.709	8.089	106.82
Des-ethyl atrazine-13C3 (Sur)	1.427	5.506	82.44
Orphenadrine (Sur)	0.562	11.559	129.59
Oleandomycin (Sur)	0.788	8.825	108.53
Ampicillin	0.980	1.036	65.34
Azithromycin	0.892	1.047	70.31
Carbadox	0.725	1.262	61.80
Ceftiofur	11.792	7.536	15.31
Ciprofloxacin	2.124	1.344	54.29
Clarithromycin	1.221	0.347	40.26
Danofloxacin	1.349	0.631	61.47
Desfuroyl ceftiofur cysteine disulfide	10.691	8.370	18.70
Erythromycin	0.457	0.583	64.84
Florfenicol	8.486	6.005	102.92
Lincomycin	0.591	0.678	12.09
Monensin	5.856	7.088	84.53
Novobiocin	87.581	nd	nd
Ormetoprim	0.348	1.276	61.65
Oxacillin	7.881	4.560	25.96
Oxolinic acid	0.356	0.644	83.99
Penicillin G	14.763	4.597	47.72
Penicillin V	116.729	8.835	49.01
Penillic acid	5.985	2.339	9.83
Roxithromycin	0.781	6.158	323.38
Sulfachloropyridazine	1.381	1.677	109.24
Sulfadiazine	0.509	2.656	144.47
Sulfadimethoxine	0.958	1.909	165.24
Sulfamerazine	0.962	2.608	152.66
Sulfamethazine	0.933	1.163	108.09
Sulfamethizole	1.668	1.287	105.21
Sulfamethoxazole	1.035	0.732	83.89
Sulfanilamide	2.366	6.218	30.52
Sulfathiazole	0.900	2.630	165.18
Thiabendazole	0.203	0.693	87.84
Tiamulin	0.405	0.826	89.26
Trimethoprim	0.874	1.449	60.96
Tylosin	1.036	0.497	79.58
Virginiamycin	7.916	65.771	295.31

Table S4. Relative abundance of the 25 most abundant bacterial orders

Order	CONTROL[a]		LO-EFF-UP[a]		LO-EFF-DN[a]		HI-EFF-UP[a]		HI-EFF-DN[a]		p value[b]
Bacteria_unclassified	14.9%	ab	18.5%	bc	12.7%	a	15.3%	ab	21.9%	c	0.000
Betaproteobacteria_unclassified	8.1%	a	9.1%	a	7.0%	ab	7.4%	ab	5.0%	b	0.001
Burkholderiales	7.7%	ab	6.2%	abc	10.8%	b	5.7%	ac	4.9%	c	0.001
Gammaproteobacteria_unclassified	4.3%	ab	4.4%	abc	6.6%	c	4.7%	bc	3.4%	a	0.001
Rhizobiales	5.4%	a	3.5%	b	6.2%	a	3.5%	b	4.5%	ab	0.002
Sphingobacteriales	4.8%	a	2.4%	b	5.6%	a	3.0%	b	4.5%	a	0.001
Bacteroidetes	3.5%	ab	4.0%	b	2.8%	a	5.2%	b	2.9%	a	0.002
Proteobacteria_unclassified	3.2%	a	4.3%	b	4.0%	ab	3.2%	ab	3.0%	a	0.006
Planctomycetales	3.4%	ab	2.0%	c	2.2%	bc	2.0%	bc	3.8%	a	0.001
Anaerolineales	1.9%	ab	4.3%	c	1.5%	b	3.0%	ac	2.0%	ab	0.000
Rhodocyclales	1.8%	ab	2.7%	bc	1.4%	a	4.0%	c	1.2%	a	0.000
Alphaproteobacteria_unclassified	2.0%	abc	0.7%	c	2.6%	b	1.6%	ac	2.4%	ab	0.000
Sphingomonadales	1.4%	ab	0.6%	b	4.0%	a	1.5%	ab	1.6%	ab	0.001
Nitrospirales	2.7%	a	0.5%	b	2.1%	a	2.5%	a	1.3%	ab	0.001
Acidobacteria Gp6	2.1%	ab	1.8%	abc	2.5%	b	1.1%	c	1.5%	ac	0.000
Myxococcales	1.3%	a	2.1%	b	1.4%	a	1.9%	ab	2.1%	b	0.001
Rhodobacterales	2.1%	ab	0.7%	c	3.0%	b	1.8%	abc	1.0%	ac	0.000
Desulfobacterales	2.7%	a	2.3%	a	0.7%	b	2.3%	a	0.2%	b	0.000
Actinomycetales	1.0%	ab	1.7%	bc	0.8%	a	1.0%	ab	3.3%	c	0.000
Xanthomonadales	1.3%	ab	1.6%	ac	0.7%	b	1.6%	ac	1.9%	c	0.001
Cytophagales	1.1%	a	1.3%	ab	1.7%	ab	1.6%	b	1.4%	ab	0.038
Flavobacteriales	0.7%	ab	0.6%	b	1.6%	abc	1.9%	ac	2.0%	c	0.003
Hydrogenophilales	1.9%	a	1.9%	a	0.8%	ab	1.9%	a	0.2%	b	0.001
Verrucomicrobia Subdivision 3	0.8%	a	1.5%	ab	0.8%	a	1.9%	b	1.0%	a	0.001
Gaiellales	1.1%	ab	1.0%	abc	0.6%	bc	0.3%	c	2.4%	a	0.000

[a] Mean values (n=5). Different letters within a row indicate significant differences between sites based on Dunn's test (p<0.05).

[b] p values based on Kruskal-Wallis test .

Table S5. AMOVA and PERMANOVA results for comparisons of bacterial community composition between sites

Site	AMOVA p-Value	PERMANOVA p-Value
Across All sites	<0.001	0.008
CONTROL - LO-EFF-UP	0.010	0.006
CONTROL - LO-EFF-DN	0.006	0.005
CONTROL - HI-EFF-UP	0.007	0.008
CONTROL - HI-EFF-DN	0.012	0.007
LO-EFF-UP - LO-EFF-DN	0.014	0.008
HI-EFF-UP - HI-EFF-DN	0.007	0.008
LO-EFF-UP - HI-EFF-UP	0.005	0.009
LO-EFF-UP - HI-EFF-DN	0.005	0.012
LO-EFF-DN - HI-EFF-UP	0.010	0.013
LO-EFF-DN - HI-EFF-DN	0.011	0.008

Table S6. Bacterial OTUs with the largest differences in relative abundance between the downstream sites on the low-effluent and high-effluent streams.

Operation Taxonomic Unit[a]	LO-EFF-DN[b]			HI-EFF-DN[b]			p-value[c]
Comamonadaceae	1.77%	+/-	0.21%	0.54%	+/-	0.03%	0.001
Gammaproteobacteria	0.96%	+/-	0.16%	0.00%	+/-	0.00%	0.001
Sphingorhabdus	1.29%	+/-	0.13%	0.36%	+/-	0.04%	<0.001
Burkholderiales	1.17%	+/-	0.15%	0.23%	+/-	0.03%	0.001
Comamonadaceae	1.76%	+/-	0.12%	0.85%	+/-	0.08%	<0.001
Rhodobacteraceae	1.35%	+/-	0.13%	0.47%	+/-	0.03%	<0.001
Rhodobacteraceae	1.00%	+/-	0.13%	0.14%	+/-	0.02%	<0.001
Gammaproteobacteria	0.83%	+/-	0.13%	0.03%	+/-	0.01%	0.001
Proteobacteria	0.79%	+/-	0.14%	0.03%	+/-	0.01%	0.001
Burkholderiales	1.44%	+/-	0.11%	0.72%	+/-	0.05%	0.001
Gammaproteobacteria	0.72%	+/-	0.01%	0.09%	+/-	0.01%	<0.001
Mycobacterium	0.03%	+/-	0.01%	0.62%	+/-	0.09%	<0.001
Proteobacteria	0.55%	+/-	0.22%	0.03%	+/-	0.01%	0.034
Sphingorhabdus	0.72%	+/-	0.10%	0.23%	+/-	0.04%	0.001
Bacteria	0.09%	+/-	0.01%	0.57%	+/-	0.03%	<0.001
Thiobacillus	0.67%	+/-	0.08%	0.19%	+/-	0.01%	0.001
Planctomycetaceae	0.00%	+/-	0.00%	0.47%	+/-	0.07%	<0.001
Novosphingobium	0.75%	+/-	0.06%	0.29%	+/-	0.01%	<0.001
Actinomycetales	0.02%	+/-	0.00%	0.47%	+/-	0.05%	<0.001
Rhizobiales	0.52%	+/-	0.05%	0.07%	+/-	0.00%	<0.001

[a] OTUs were assigned to the lowest possible taxonomic rank and are listed in order of decreasing difference in relative abundance between the two sites.

[b] Mean values +/- standard error (n=5).

[c] Based on one way ANOVA
